# Experiences of Multidisciplinary Development Team Members During User-Centered Design of Telecare Products and Services: A Qualitative Study

**DOI:** 10.2196/jmir.3195

**Published:** 2014-05-19

**Authors:** Joan Vermeulen, Renée Verwey, Laura MJ Hochstenbach, Sanne van der Weegen, Yan Ping Man, Luc P de Witte

**Affiliations:** ^1^CAPHRIDepartment of Health Services ResearchMaastricht UniversityMaastrichtNetherlands; ^2^Research Center for Technology in CareFaculty of Health CareZuyd University of Applied SciencesHeerlenNetherlands

**Keywords:** user-centered design, telecare, eHealth, participation, multidisciplinary team, barriers and facilitators

## Abstract

**Background:**

User-centered design (UCD) methodologies can help take the needs and requirements of potential end-users into account during the development of innovative telecare products and services. Understanding how members of multidisciplinary development teams experience the UCD process might help to gain insight into factors that members with different backgrounds consider critical during the development of telecare products and services.

**Objective:**

The primary objective of this study was to explore how members of multidisciplinary development teams experienced the UCD process of telecare products and services. The secondary objective was to identify differences and similarities in the barriers and facilitators they experienced.

**Methods:**

Twenty-five members of multidisciplinary development teams of four Research and Development (R&D) projects participated in this study. The R&D projects aimed to develop telecare products and services that can support self-management in elderly people or patients with chronic conditions. Seven participants were representatives of end-users (elderly persons or patients with chronic conditions), three were professional end-users (geriatrician and nurses), five were engineers, four were managers (of R&D companies or engineering teams), and six were researchers. All participants were interviewed by a researcher who was not part of their own development team. The following topics were discussed during the interviews: (1) aim of the project, (2) role of the participant, (3) experiences during the development process, (4) points of improvement, and (5) what the project meant to the participant.

**Results:**

Experiences of participants related to the following themes: (1) creating a development team, (2) expectations regarding responsibilities and roles, (3) translating user requirements into technical requirements, (4) technical challenges, (5) evaluation of developed products and services, and (6) valorization. Multidisciplinary team members from different backgrounds often reported similar experienced barriers (eg, different members of the development team speak a “different language”) and facilitators (eg, team members should voice expectations at the start of the project to prevent miscommunication at a later stage). However, some experienced barriers and facilitators were reported only by certain groups of participants. For example, only managers reported the experience that having different ideas about what a good business case is within one development team was a barrier, whereas only end-users emphasized the facilitating role of project management in end-user participation and the importance of continuous feedback from researchers on input of end-users.

**Conclusions:**

Many similarities seem to exist between the experienced barriers and facilitators of members of multidisciplinary development teams during UCD of telecare products and services. However, differences in experiences between team members from various backgrounds exist as well. Insights into these similarities and differences can improve understanding between team members from different backgrounds, which can optimize collaboration during the development of telecare products and services.

## Introduction

The number of people who suffer from chronic conditions and the number of elderly people are increasing, and at the same time, the number of care professionals is decreasing [1]. The gap between the demand for and supply of care that results from these changes will put a burden on patients, care professionals, and health care systems in the near future [2,3]. Telecare products and services have the potential to alleviate this burden by supporting self-management, remote monitoring of health conditions, and the delivery of interventions [4-6]. The appropriate adoption of telecare technologies can contribute to the lives of patients with chronic conditions and elderly people by improving their quality of life and enabling them to live independently for as long as possible [7,8].

Unfortunately, previous studies show that telecare does not always fulfill its potential because the telecare products and services that are developed do not fit the needs and preferences of end-users or because they do not fit the context in which they should be implemented [9-12]. Involving end-users in the development of telecare products and services can ensure that human and non-technology issues are taken into consideration, which improves the usability and acceptability of the technology that is being developed [13-15].

Theoretical frameworks regarding user-centered design (UCD) have been used as guidelines during the development processes of telecare products and services [16-22]. Such frameworks take into account important key principles of UCD, for example, the development process should be iterative and incremental, the end-users should be actively involved from early development stages onwards, design options should be explained to end-users in a language that they understand, the developed services and products should be evaluated in a real life context, and the development process should be performed by effective multidisciplinary teams [23]. Previous studies revealed that various barriers and challenges can occur during UCD processes that can influence the collaboration between multidisciplinary stakeholders. Examples of such barriers are communication between team members from different backgrounds, management of expectations, and availability of recourse for the involvement of users [14,24,25].

Understanding how members of multidisciplinary development teams with different backgrounds experience the UCD process might help gain insight into factors that different members consider critical during the development of telecare products and services. Furthermore, it might reveal how they deal with barriers and challenges they encounter. Therefore, the primary objective of this study was to explore how members of multidisciplinary development teams experienced the UCD process of telecare products and services. The secondary objective was to identify differences and similarities in the barriers and facilitators they experienced.

## Methods

### Design and Participants

This qualitative study was conducted in parallel with four (subsidized) Research and Development (R&D) projects that were initiated by Maastricht University and/or Zuyd University of Applied Sciences. The projects were selected because they developed a variety of telecare products and services to support self-management in different user-groups: patients with diabetes or chronic obstructive pulmonary disease (COPD) [26-28], patients with cancer pain, frail elderly people, or elderly people living at home [29]. A prerequisite for project selection was that end-users were considered official members of the development team. Multimedia Appendix 1 provides information regarding the four projects. Principle investigators of the projects had a background in health sciences.

For this study, a purposeful sample of the “core members” of the four development teams, who were involved throughout the whole development process from the beginning onwards, were invited to participate in a semistructured interview. Principle investigators of each development team identified the core members in consultation with their team and asked them whether they would like to receive an invitation for this study via email. The 25 core members were identified across the four R&D projects: 3 elderly persons, 2 professional advisors of these elderly persons (specialized in facilitation participation of elderly persons during research projects), 2 patients with chronic conditions, 1 geriatrician, 2 nurses, 5 software/technical engineers, 4 managers of R&D companies or engineering teams, and 6 researchers/principle investigators of the R&D projects. All these potential participants received an invitation letter via email from the researcher (JV) explaining the purpose and details of this qualitative study. All invitees accepted the invitation.

### Procedures

After invitees agreed to participate, the interview was scheduled at a place and time that was convenient for the participant. As a result, 13 interviews were conducted at the university, nine at the workplace of the participant, and 3 via Skype/telephone call. The 6 participants of this study who were researchers or principle investigators in one of the four R&D projects (JV, RV, LMJH, SvdW, YPM, LdW) were interviewed by an experienced external interviewer who was not involved in any of the R&D projects described above. JV, RV, LMJH, SvdW, and YPM interviewed the remaining 19 participants of this study. All participants were interviewed by a researcher who was not involved in the same R&D project as the participant. Interviews with members of development teams of Projects 1, 3, and 4 were conducted when final products were already developed. Interviews with members of the development team of Project 2 were conducted at a prototype stage. Interviews were recorded using a digital voice recorder and lasted between 35 and 65 minutes. Average duration of an interview was 46 minutes.

All semistructured interviews were based on the following topic list: the role of the participant in the project, the aim of the project, experiences during the development process, points of improvement for the development process, and what the project meant to the participant. This topic list was established before the start of the study and was not changed during the course of the study. Interviewers used reflective listening techniques to encourage participants to elaborate on their views. By reflecting back to the participants during the interview what the interviewer believed was said, the statements of the participant were verified and/or clarified. By keeping an open posture and by emphasizing at the beginning of the interview that there were no right or wrong answers, participants were encouraged to share their own personal opinions and thoughts. All participant answers were kept confidential. To guarantee anonymity, the following terms are used in this article to refer to the data obtained from certain groups of participants:

- “End-user” refers to data obtained from an elderly person, an advisor of an elderly person, or a patient with a chronic condition (n=7).
- “Professional end-user” refers to data obtained from a geriatrician or a nurse (n=3).
- “Engineer” refers to data obtained from a software/technical engineer (n=5).
- “Manager” refers to data obtained from a technical project leader or owner of an R&D company (n=4).
- “Researcher” refers to data obtained from a researcher or principle investigator of the R&D projects (n=6).

### Data Analyses

Once all interviews were conducted, they were transcribed verbatim by two of the researchers (JV/RV) or a research assistant. Afterwards, JV checked the transcripts against the audio recordings. All transcripts were coded using NVivo version 9.0. Field notes from the interviews were also included in the analyses if they were available. Two researchers (JV & RV) started analyzing the data using a conventional content analysis approach. They independently coded six transcripts of interviews that were conducted with members from different development teams with different backgrounds. After initial coding, the 2 researchers checked for consensus, and after discussion, they agreed on the main themes and subthemes of the coding scheme. This coding scheme was used by JV to analyze the remaining interviews. If in doubt about whether data from these remaining interviews fitted the coding scheme or not, JV consulted RV. Themes and subthemes were refined or extended based on the data from the remaining interviews to be analyzed, and if necessary, new (sub)themes were added. Once all transcripts were analyzed, the content of the themes and subthemes of the coding scheme were discussed with the research group that included all co-authors of this paper. Consensus was reached on themes that were related to different phases of the development process and subthemes that related to barriers and facilitators participants experienced during these phases. After completing the data analyses, findings related to themes and subthemes were reported back to all participants for a member check.

## Results

### Themes

The main themes that emerged from the analyses related to different phases of the UCD process were (1) creating a development team, (2) expectations regarding responsibilities and roles, (3) translating user requirements into technical requirements, (4) technical challenges, (5) evaluation, and (6) valorization. Experiences of participants during these different phases are described below. Tables 1 and 2 provide an overview of which experienced barriers and facilitators respectively were reported by which members of the development team, to gain insight into differences and similarities.

**Table 1:** Experienced barriers in the UCD process of telecare products and services according to different members of the development team.

Theme	Barriers	Participant groups^a^				
		EU	PU	EN	MA	RE
Creating a development team	Team members come from different backgrounds and therefore do not speak the same “language” (use different terminology).	X	X	X	X	X
Expectations regarding responsibilities and roles	Team members have different implicit expectations regarding project management, tasks of team members, and delivery of content (especially at the start of the project).	X	X	X	X	X
Translation of user requirements into technical requirements	Prioritizing user-requirements with various stakeholders is more time consuming than expected.			X	X	X
	Iterative adaptations of user-requirements (especially in later stages) place a serious strain on the budget/time of the project.			X	X	X
Technical challenges	Integration of different technologies or platforms into one telecare service is difficult (but necessary).	X	X	X	X	X
	Time allowed for telecare development is short in subsidized projects which causes problems with robustness in the real life setting and large scale evaluation research.					X
	The commercial market is developing similar products at a rapid pace which makes it difficult to keep up.	X		X	X	X
Evaluation	Members of the development team are not the best evaluators because they find their own “work-arounds” to avoid bugs (unaware).			X		X
	Recruitment of patients and professionals for the longitudinal evaluation of the developed telecare products/services is time-consuming.	X	X	X	X	X
	Too many different projects/devices are offered to potential end-users at the same time.				X	X
Valorization	Different partners/companies who are involved have different ideas about what makes a good business case.				X	

^a^EU=end-users, PU=professional end-users, EN=engineers, MA=managers, RE=researchers.

**Table 2:** Experienced facilitators in the UCD process of telecare products and services according to different members of the development team.

Theme	Facilitators	Participant groups^a^				
		EU	PU	EN	MA	RE
Creating a development team	Team members recognize their complementary knowledge and skills, which creates a team spirit.	X	X	X	X	X
	Team members agree that end-users should be involved in the development team from the beginning of the development process.	X	X	X	X	X
	Project leaders and managers advocate that input of all team members should be treated as equal.	X				
	Researchers report back to end-user representatives about why their advice was followed or not.	X				
	Researchers visit team members at home/work (when team meetings are not possible due to differences in schedules).	X	X	X		
Expectations regarding responsibilities and roles	Team members voice expectations at the start of the project to prevent miscommunication at a later stage (but this may be difficult due to the fact that many expectations are implicit).	X	X	X	X	X
Translation of user requirements into technical requirements	Engineers help researchers to translate user-requirements into technical requirements to speed up this process.			X	X	X
Technical challenges	Researchers should take enough time to conduct small scale usability tests and pilot studies before moving to large trials to improve technical functioning and robustness.					X
	Products/services developed in the projects are easier to integrate in care processes compared to off-the-shelf products.	X	X	X	X	X
Evaluation	Members of the development team evaluate prototypes in lab to identify bugs and/or gain insight into experiences with the products/services.	X		X		X
	Care professionals receive reimbursement for the increased workload that comes with participating in an evaluation study.		X			X
	Study participants can be recruited via the network of the members of the development team, especially via patient/elderly representatives.	X				X
Valorization	Allocate part of the budget to the development of a business case and start with this at the beginning of the project.			X	X	

^a^EU=end-users, PU=professional end-users, EN=engineers, MA=managers, RE=researchers.

### Creating a Development Team

Due to the fact that members of the development teams had different backgrounds, there was also a difference in knowledge about the project, UCD processes, telecare products and services, intended end-users of the technology, and ways of conducting research in a health care setting. In addition, different members of the development team seemed to speak a “different language” (use different terminology):

We have researchers, we have doctors, we have technicians, and it is very, it is not the same world. Because we do not speak the same language…Sometimes we think something and for example the researcher understands something else…We speak technical, she (the researcher) talks with elderly people, she talks with other worlds. That is why it is so difficult.

P20, male, engineer

Participants reported that overcoming these differences was sometimes difficult. A benefit of these differences, mentioned by most participants, is that members of the team could really complement each other, which resulted in a positive “team spirit”:

They (technical engineers) did not know anything about medication and then you think; well I don’t know anything about computers. So together that was fun, as if you speak a different language but still have to come up with a solution together.

P23, female, professional end-user

Multidisciplinary collaboration was positively influenced by the fact that patients and care professionals were involved and recognized as members of the development teams from the start. When managers or supervisors advocated that the input of all members should be treated as equal, this facilitated co-creation and UCD, according to end-users. Furthermore, end-users indicated that they appreciated it when the researcher reported back to them about which parts of their advice were followed and which were not. According to some, this feedback was more important than actually following their advice because it made them feel appreciated as a team member:

I did have the feeling that, the things that we put forward, that they (the researchers) did something with that. And sometimes they (the researchers) just said: “listen, we did not choose this or we did choose this”. And I think that is important…Feedback is very important. For example when you put something forward and after that you don’t hear anything. Then you don’t know whether something was done with it at all or whether it was taken seriously. And then it is also difficult to be in that process together.

P9, female, end-user

Finally, the organizational aspect of working in multidisciplinary teams was a challenge in most projects. Since most members had different schedules, it was often difficult to organize meetings with the whole team. Submeetings were a satisfactory solution according to most. Elderly representatives, care professionals, and engineers indicated that they appreciated the flexible attitude of researchers who visited them at home or at work to offer additional information/explanation or an update.

### Implicit Expectations Regarding Responsibilities and Roles

Participants indicated that during the project they discovered differences in expectations regarding responsibilities and roles between members of the development team. Examples of issues where expectations differed between members of the development team were who the project manager is, what the tasks are of different team members, and who delivers content of the services that are being developed. Participants seemed to agree that clearly expressing and communicating expectations at the start of the project would help to ensure that the entire development team was on the same page, which would probably optimize multidisciplinary collaboration. However, some participants experienced that voicing these expectations could be difficult since they are often implicit:

Look, in fact you start this project with an open mind and you create the expectations along the way. Because we work in a certain way: iterative design. We take it a step further every time. So, at the beginning of the project you actually do not know where you will end up. You work towards the end gradually. So, you actually do not have very detailed expectations before the start.

P15, male, engineer

I expected more vision, more strategy, a clearer picture about this (concept of the project) at the start of the project. Well that disappointed me. That fit with the patient group was created along the way. I would have liked to feed of the available knowledge of the university. But that was disappointing.

P16, male, manager

I think that we had a very large part in delivering content (for the telecare product and service)…Of course, you have to partly develop the intervention. But this detailed, no I didn’t expect that I would do that. I expected another share of the company and thought that was disappointing in hindsight.

P2, female, researcher

### Translating User Requirements Into Technical Requirements

Participants experienced that collecting user requirements and discussing with the development team which requirements deserved priority took longer than expected. One reason for this is that reaching a consensus with a team consisting of members with different backgrounds can be quite challenging.

Another time-consuming part of the process was translating user requirements into the very detailed technical requirements that engineers need to be able to develop the first prototypes. Help from the engineers in this translation appeared to be crucial since most researchers did not have a technical background and were not familiar with the technical language that is used to describe technical requirements:

In the concept phase, everything seems possible. But when you have to specify things until the final feedback message, it is very difficult. And it takes a lot of time to think these things through.

P5, female, researcher

The researchers are more of less forced into a role in which they have to think along in a technical manner. And they are not used to that, it is not their job. And that creates a certain type of tension. Because they are forced to think about (technical) things that they had not thought about before.

P18, male, engineer

Finally, the identified user requirements evolved during the project as a result of the iterative nature of the UCD processes. Members of the development team agreed that these iterations were necessary to ensure that the developed products and services meet the requirements of the end-users (in the best possible way). Especially engineers and managers pointed out that they tried to be as flexible as possible in incorporating the new and additional requirements in new prototypes because they recognized the importance of the iterations. However, at a certain point, this flexibility ends because deadlines have to be met and personnel will be deployed in other projects after these deadlines:

It is good to get feedback from your target group but it is important to stay in control…I had the feeling that there were too many changes in response to feedback of the target group…At a certain time we froze the specifications and started working with that.

P18, male, engineer

We had a lot of backwards and forwards and changing. What might be a relatively simple change for an end-user, for instance the change of a bar from one place to another, could take a significant amount of time or require a major change in the way a software program was running.

P19, male, manager

### Technical Challenges

#### Overview

The technical challenges that the development teams were faced with related to the integration of different technologies or platforms, robustness of technology in a real-life setting, and rapid pace of developments on the commercial market.

#### Integration of Different Technologies or Platforms

The telecare products and services that were developed during the projects all required the integration of various technological components (eg, integration of a sensor with an application on a mobile device and an online database). Technical and software engineers, and to a lesser extent also other participants, experienced that the integration of these components was often more difficult than expected for several reasons. First, engineers experienced that the components sometimes have their own “language” and specifications (eg, hardware vs software), which makes integration more complicated. Second, the input for the system as a whole, and input regarding the integration of the developed technology in care, was provided by different members of the development team. All team members recognized that integrating the input from these parties into the products and services can be a real challenge as it does not always match. Third, in some cases the different components are not finished at the same time. This can seriously delay the progress of projects.

Every company developed their part (of the technology)…We develop something nice but then it does not fit. And then the other company develops something nice and our system cannot handle it. And then the researchers provide a new part of the content and then we think: where can we put this? That was difficult to work with sometimes. We resolved it in the end but I think things could have run more efficient in some areas.

P17, male, engineer

#### Robustness of Telecare Products in Real-Life Setting

Researchers experienced that there was often not enough time and budget for the development of the telecare products because funding bodies assume that the technology already exists. Technology development in these projects appeared to be more challenging since the health care setting was taken into consideration, which is often not the case in existing off-the-shelf telecare products. As a result, the technology was sometimes not “mature” enough when projects moved to large trials or evaluation studies, which caused problems regarding robustness. All members of the development teams agreed that robustness is an absolute precondition for the uptake of telecare products and services in practice. Taking enough time for conducting in-lab usability tests and pilot studies before moving to real life settings might prevent problems concerning robustness in large trials according to researchers:

Funding agencies for research often assume that the technologies already exist. However, there is hardly any time for the development of the technology. But we know from experience that this is very difficult and time consuming. We should not jump to large evaluation studies too fast but first do pilots and usability evaluations before we start with the big works. That is something that is often underestimated.

P6, male, researcher

#### Developments on the Commercial Market

Many participants experienced that the commercial market sets the standard for products developed in the R&D projects; the user requirements for the telecare products and services are often influenced by what is already on the market. According to members of the development teams, it is nearly impossible to keep up with the rapid pace at which the commercial market is developing. The reason the development teams chose to create something new instead of buying off-the-shelf products is that newly developed products can be adapted to fit the health care context and end-user needs.

### Evaluation

#### Overview

In all projects, the developed devices were tested in the lab and in real-life. Issues that relate to the evaluation of the devices in both contexts are described below.

#### Evaluation in Lab

The developed products were first tested extensively by the members of the development team. Researchers who tested the products indicated that this was not only important for identifying bugs and errors but that it also provided better insight into experiences expected with end-users of the product. However, researchers and engineers indicated that eventually they were less able to identify bugs, as they unconsciously created their own “work-arounds”:

Once you actually use it (the technology) yourself, then you really bond with it so to speak. And then you become even more alert to improvements

P2, female, researcher

#### Evaluation in Real-Life

Participants experienced that the recruitment of patients and professionals who were willing to evaluate the developed products and services was a true bottleneck in the planning of the projects. A possible explanation for this problem, according to the participants, was that usually care professionals have to perform the activities for such R&D projects (eg, providing input for requirements or recruitment of patients) on top of their regular activities, which causes an increased workload. In some projects, the professionals did not get reimbursed for recruiting participants. Another problem described was that there were too many different (telecare) research projects in one area at the same time. Possible solutions for these problems, employed during the R&D projects, were reimbursing care professionals, recruiting participants via the (in)formal network of members of the development team, or organizing meetings with professionals who participate in evaluation studies to increase their awareness and create commitment to the project:

Having patience is very important in research, especially when it concerns including patients in your study. You will come across barriers, it is just like hurdling in a sense.

P24, male, professional end-user

Convincing people who have to do it alongside their job. They are doctors, and this comes on top of it…Yes, I think that people are bombarded with something new every time: another technique that would be nice. That makes it pretty difficult.

P14, male, manager

### Valorization

Once the effects of the developed telecare products and services have been evaluated, the subsidized R&D projects will end. Members of all development teams emphasized the importance of scaling up, and valorization of, the developed telecare products and services:

That is one of the problems; it will have to happen on a larger scale in the future. For that you do need a business model. Just doing projects for the sake of doing projects and doing something else when the funding ends does not make sense.

P22, male, manager

Managers from the R&D companies and some of the technical engineers indicated that it was too bad that there was little time or budget to focus on the development of a proper business model during the projects. The fact that different companies and organizations are often involved in the delivery of one telecare product and/or service is an important issue in the development of such business models and therefore a concern to most of the managers:

At the basis, you have to start thinking who will be the owner and who will make money on this…Somehow you have to figure out a model that covers the costs and leaves something extra because we are not the type of entrepreneur that keeps investing in something that does not make any money. So I do see possibilities, but it has not been defined clearly yet.

P14, male, manager

Elderly people, patients with a chronic disease, care professionals, and researchers also indicated that they had concerns regarding the upscaling of the developed products and services. Their concerns focused on questions such as who would pay and sell the products/services, how much they should cost, and how roles and responsibilities of patients and professionals would change when telecare products and services were implemented in practice:

I am afraid that it will not be financially viable. Because physiotherapy is not included in the basic health insurance which makes your target group very small.

P7, male, end-user

I think that we still have to think about that, make agreements about when you reply or do not reply (to the patient after a certain signal from the telecare product), better agreements on how you communicate with the patients (using the new telecare services).

P25, male, professional end-user

## Discussion

### Principal Results and Comparison to Previous Research

The current study provides insight into how members of multidisciplinary development teams experience the UCD process of telecare products and services. Several barriers and facilitating factors were experienced that can influence the UCD process according to different members of four multidisciplinary development teams. Most barriers that were identified were in line with previous research [14,24,25]. Some of these factors were reported by participants from different backgrounds, whereas others seemed to be more specific for a group of participants who share a similar background or role in the UCD process. Furthermore, this study also provided insight into how members of development teams tried to deal with barriers that they encountered and which actions they undertook to facilitate the development process.

All participants experienced that differences in background can cause a language gap between members of the multidisciplinary development team that can negatively affect the development process. Other researchers and designers who have recognized this barrier recommend the use of personas, scenarios, mock-ups, or prototypes to reduce the language gap [13,21,30,31]. These techniques were also used during the R&D projects that were the focus of this study. In addition, this study revealed that the gap between members of a development team can be bridged by emphasizing the way that team members can complement each other through their differences, by principal investigators who explicitly advocate equality of team member’s input, and by providing feedback to (professional) end-users regarding their input from the start onwards, throughout the development process.

Participants with different backgrounds all recognized that managing expectations regarding responsibilities and roles is a critical factor during the UCD of telecare products and services. Previous research by Gasson suggests that managing expectations regarding work roles and tasks is a critical issue in any UCD process [32]. The results of the present study revealed that although all members of the development team had read and agreed on the same project plan and UCD methodology to be used, differences in expectations still existed. Goodman-Dean et al explain that despite agreeing on the same basic nature of the UCD process, differences can still exist between the approaches of the team members [33]. Explicitly voicing detailed expectations at the start of the development process might prevent delay later in the project. However, participants of the current study recognized that this might be difficult to do. Previous studies have emphasized that project management should identify and allocate responsibilities, tasks, and roles [16,32,34]. Project managers should facilitate the UCD process in this way without being overly prescriptive or bureaucratic since that might impede the creative nature of the design process [34].

The results of this study confirmed that time- and budget-related issues seemed to play an important role during different phases of the UCD process. The main time constraining factor reported in previous studies is that working with users in an iterative way takes too much time, regardless of the methodology used [13,14,34,35]. A literature review by Shah et al suggests that the lack of available end-users is a barrier during the UCD process [14]. The findings of the current study are not entirely in line with this since recruiting users to be involved in the development team and to test prototypes during the iterative UCD process seemed a lesser barrier compared to recruiting participant for longitudinal research. A possible explanation for this could be that for the prior UCD activities, participants were recruited via the (informal) network of end-users and professional end-users who were part of the development team. Through this, the involvement of (professional) end-users from early stages onwards facilitated the progress of the projects. Other studies that have been conducted previously did not indicate which factors were considered too time-consuming. The current study provides insight into causes of time-related barriers as experienced by different members of multidisciplinary development teams. Researchers and engineers were the groups of participants who most frequently reported on time-related barriers and how they tried to deal with them.

A critical factor reported on by all managers is the development of a good business model. Results of this study revealed that budget should be allocated to the development of a business case and that the stakeholders involved should discuss business modeling issues at an early stage. Previous studies have emphasized that business model development should run in parallel with the UCD of eHealth technologies because it contributes to the development of such technologies [16,36].

### Strengths and Limitations

The scope of the four R&D projects included in this study varied from general services related to care and well-being for elderly people living in the community to specific services for severely ill patients who suffer from cancer (treatment) pain. The variety of projects included increases the generalizability of the findings. Furthermore, all core members of the multidisciplinary development teams were interviewed in order to incorporate all points of view in our analyses. This is a strength of the study design since previous studies aimed at identifying barriers of UCD processes often focused merely on the perspective of the designers or developers [13,33,36]. Including participants from different backgrounds created triangulation of data resources, which increased trustworthiness of the findings. Furthermore, the member check revealed that participants agreed with the experienced barriers and facilitators that were identified.

When interpreting the results of this study, some limitations of the study design should be taken into account. First, in the interviews, participants reflected on development processes that started 1-2 years ago, which might have caused some degree of memory bias. However, reflecting on the development process at later stages might also have benefits over interviewing participants in the middle of the development process. In the latter situation, the answers of participants may be influenced by the stage of the project at the time of the interview. Second, participants were asked to reflect critically upon development processes in which they were involved, and consequently they had to reflect on their own actions and the actions of their team members, which could have been a sensitive topic. In order to limit this sensitivity, a researcher who was not part of the participant’s development team conducted the interview. However, it cannot be ensured that all barriers and facilitators were reported to the interviewers. Third, the authors of this paper had a double role in this study since they interviewed the participants who were not researchers, but they were also core members of the development teams (and hence were interviewed themselves). The involvement and experiences of the researchers with UCD of telecare products and services could have influenced data collection and/or analyses. We aimed to minimize these influences by letting researchers interview only participants who were involved in different R&D projects than they were, by developing a topic list for the interviews that was used by all interviewers throughout the study, and by developing the coding scheme with 2 researchers. Finally, data from the interviews with the researchers and from the interviews with other team members were treated as equal in the analyses. This is not necessarily a limitation, but it might be considered a notable feature of the study design. The main reason for this novel and somewhat unusual approach was that the researchers themselves had experienced and influenced the development process just as much as other members of the development team. Not interviewing the researchers could have resulted in missing barriers and facilitators that they themselves experienced.

### Conclusions

Many similarities seem to exist between the barriers and facilitators experienced by members of multidisciplinary development teams during UCD of telecare products and services. However, differences in experiences between team members with different backgrounds exist as well. Insights into these similarities and differences can improve understanding between team members from different backgrounds, which can optimize collaboration during the development of telecare products and services.

## Multimedia Appendix 1

Description of four R&D projects developing telecare products and services.

## Abbreviations

R&D: research and development
UCD: user-centered design
