# Supplementary material for: Experiences of Multidisciplinary Development Team Members During User-Centered Design of Telecare Products and Services: A Qualitative Study
Source: J Med Internet Res. 2014 May 19;16(5):e124. doi: 10.2196/jmir.3195 (PMC4051739; doi:10.2196/jmir.3195)
Supplement: Supplementary file 1 [file jmir_v16i5e124_app1.pdf]

Appendix 1: Description of four R&D projects developing telecare products and services.

**Project 1: It's LiFe! [26-28]**	
**Aim of the project**	Development and evaluation of a monitoring and feedback tool to support self-management through lifestyle feedback for patients with chronic diseases in primary care.
**Intended end users**	• Diabetes and COPD patients<br>• Practice nurses
**Telecare products and services developed**	The system measures physical activity with an activity monitoring sensor and gives feedback and dialogue sessions, based on a personal activity goal, on a smartphone-based application and website. The goal is set in minutes a day by the care professional together with the patient. Care professionals can track the progress of their patients via a web-based monitoring system, in which the data from patients appears automatically.
**Intended outcome**	Increased daily physical activity levels.
**Involved stakeholders in UCD process**	• 6 researchers with different backgrounds (health sciences, medicine, nursing, movement sciences, and psychology).<br>• 8 technical experts (2 technical project leaders and 6 engineers).<br>• 2 patient representatives with chronic conditions.
**Phases of UCD process and methods used**	Four phases:<br>1) Identify end-users and context<br>• Literature search to identify users and context.<br>2) Concept development<br>• Literature and experts consultation to set up a use case.<br>3) Tool (Re) design<br>• Interviews with 15 patients and 16 care professionals/experts to identify user requirements.<br>• Focus group interviews with patients to check user requirements.<br>• Expert meeting.<br>4) Evaluation of the prototype in lab and in real-life.<br>• Heuristic evaluation.<br>• In lab usability test of smartphone-based application by patients with diabetes or chronic obstructive pulmonary disease (COPD).<br>• Usability test of computer-based monitoring system by practice nurses.<br>• Pilot-study in primary care setting.
**Project 2: Self-management support for patients with cancer pain**	
**Aim of the project**	Development and evaluation of a technology supported self-management intervention for outpatients with cancer (treatment) related pain.
**Intended end users**	Patients with cancer pain and nurses specialized in pain and palliative care.
**Telecare products and**	The intervention includes an iPad application for patients that

**services developed**	is connected to a web application for nurses. Patients monitor their pain, symptoms and medication use daily. Based on these registrations they are provided with graphical feedback information and educational sessions. Nurses remotely keep track on patient data and provide patients with advice, while collaborating with the treating physician and pharmacist.
**Intended outcome**	Lower pain intensity scores and a better quality of life.
**Involved stakeholders in UCD process**	• 3 researchers with different backgrounds (health sciences, pain, palliative care, and telecare).<br>• 3 technical experts (1 designer, 1 software engineer, and 1 specialist in telemedicine).<br>• 3 care professionals (1 pain/palliative care specialist and 2 pain/palliative care nurses).
**Phases of UCD process and methods used**	Three iterative phases:<br>In each of these phases a sequence of five iterative steps was performed: research, ideas, prototyping, evaluation and documentation.<br>1) Exploration of context<br>• Document analysis (guidelines, case reports) to explore patient characteristics and usual care in the outpatient setting.<br>• Semi-structured interviews (10 care professionals, 2 patients) to identify intervention needs.<br>2) Specification of content<br>• Document analysis (pain anamnesis protocols, education materials) and 3 consultation sessions with a multidisciplinary palliative care team to provide input for the different intervention components.<br>3) Organization of care<br>• Literature review to provide insight into the presentation of information within the application.<br>• Semi-structured interviews with 4 care professionals to setup the care organization in which the intervention was to be embedded.<br>After these three phases, usability and feasibility of the telecare products and services were evaluated by patients and nurses in an in-lab usability test and in a real-life pilot study.
**Project 3: Telecommunication technology supporting care and wellbeing**	
**Aim of the project**	Development of a telecommunication portal that can assist frail elderly people in independent living.
**Intended end users**	Frail community-dwelling elderly people.
**Telecare products and services developed**	The telecommunication portal is provided to the users via a touch-screen computer. It integrates several functionalities, such as: access to information and remote communication between the elderly person and their environment (e.g. family, informal caregivers, healthcare and welfare services). It supports frail elderly people in living independently at home, with social participation in the community, their wellbeing, and asking for healthcare services when necessary.

**Intended outcomes**	Increased independence.
**Involved stakeholders in UCD process**	• 2 researchers with different backgrounds (health sciences and medical technology).<br>• 6 technical experts (2 technical project leaders and 4 software engineers).<br>• 4 elderly user representatives and 1 advisor of these representatives.
**Phases of UCD process and methods used**	Seven phases:<br>1) Identification of end-users<br>• Literature search and expert consultation to identify users and context.<br>2) Selection of end-users<br>• Experts and elderly representatives were consulted.<br>3) Needs assessment among end-users<br>• 3 participatory observations with frail elderly persons.<br>• 14 semi-structured interviews with frail elderly persons.<br>• Set up of 5 use cases to discuss in focus groups.<br>• 2 focus group interviews with frail elderly persons.<br>4) Identification of needs among healthcare and welfare services<br>• Healthcare and welfare services were consulted to discuss the requirements for the innovation.<br>5) Development of a prototype and evaluation<br>• Regular meetings and contact between the researchers, relevant stakeholders (care professionals, representatives of welfare services and elderly representatives) to discuss the requirements for the innovation.<br>6) Optimization of the prototype and evaluation<br>• Heuristic evaluation and usability test of prototype by experts and elderly representatives.<br>7) Evaluation of the effects in real field<br>• Usability and feasibility test of the prototype in a pilot study.
**Project 4: Monitoring of physical frailty in elderly people [29]**	
**Aim of the project**	Development of a monitoring and feedback system that community-dwelling elderly people can use to monitor indicators of physical functioning that are predictors of disability.
**Intended end users**	Community-dwelling elderly people.
**Telecare products and services developed**	The monitoring and feedback system consists of a bathroom scale that can measure weight and balance, a Grip-ball that can measure grip strength, and a mobile phone that can measure physical activity using a built-in accelerometer. All measurements are automatically forwarded to the mobile phone using Bluetooth. Via the interface of the smartphone feedback is provided to the user regarding (changes in) their physical functioning. Via the mobile phone, the data can be

	forwarded to a database that is accessible for care professionals.
**Intended outcome**	Increased self-management.
**Involved stakeholders in UCD process**	• 5 researchers with different backgrounds (medicine, psychology, health sciences, epidemiology, physiotherapy).<br>• 4 technical experts (1 technical project leader, 2 software engineers, and 1 web designer).<br>• 3 elderly user representatives and 1 advisor of these representatives.
**Phases of UCD process and methods used**	Five phases:<br>1) Selection of users<br>• Elderly representatives volunteered to be part of the development team.<br>2) Analysis of users and their environmental context<br>• Literature search.<br>• Four discussion group meetings with care professionals.<br>3) Identification of user requirements<br>• Three user-group meetings with elderly representatives and their advisor.<br>• Workshop with community-dwelling elderly people.<br>4) Development of a prototype of the interface (and verification with user requirements)<br>• Verification of first prototype by elderly representatives.<br>5) Evaluation of the prototype of the interface (and adaptation to user requirements)<br>• Heuristic evaluation of prototype by experts and non-experts.<br>• Usability test of prototype in lab via think aloud procedure.<br>• Usability and feasibility test of prototype in pilot study.
